# Successfully cured a rare case of esophageal squamous cell carcinoma combined with hepato-gastric schwannoma using robot-assisted surgery: case report

**DOI:** 10.3389/fonc.2025.1589929

**Published:** 2025-06-26

**Authors:** Qun-Xian Zhang, Qiang Guo, Hua Liu, Jun Zhou, Jia-Long Guo, Jun Zhang

**Affiliations:** Department of Cardiothoracic Surgery, Taihe Hospital, Hubei University of Medicine, Shiyan, China

**Keywords:** esophageal squamous cell carcinoma, schwannoma, lymph nodes, robot-assisted surgery, computed tomography

## Abstract

Esophageal squamous cell carcinoma (ESCC) is among the most common malignant tumors. Robot-assisted surgery using the cervical and thoracoabdominal incisions is a viable treatment option for ESCC. This case report presents a rare instance of ESCC complicated by hepato-gastric schwannoma, offering clinical insights and treatment strategies. A 69-year-old patient diagnosed with ESCC via gastroscopy was admitted without complaints of dysphagia. Gastroscopy revealed swollen erosions approximately 32–38 cm from the incisors. Pathology confirmed high-grade intraepithelial neoplasia and squamous cell carcinoma (SCC) of the esophageal squamous epithelium. Endoscopic ultrasonography revealed a lesion with moderate to low echogenicity, indicating local involvement of the muscularis propria. An enhanced upper abdominal computed tomography (CT) scan showed a 3.3 × 2.6 cm mass in the hepatogastric space, accompanied by mildly enlarged lymph nodes—the largest measuring 0.8 cm—which suggested possible metastasis. After a comprehensive evaluation, the patient underwent robot-assisted thoracoscopic partial esophagectomy, intrathoracic esophagogastric anastomosis, thoracoscopic adhesiolysis, and mediastinal lymphadenectomy under general anesthesia. Postoperative pathology showed a poorly to moderately differentiated ESCC (pT_1a_N_0_M_0_) measuring 3 cm × 2.7 cm × 0.2 cm, invading the lamina propria without vascular or neural invasion, and there is a schwannoma with a diameter of 3.5cm. No cancer was found at the gastric margin or anastomotic stump. Lymph nodes—including right and left recurrent laryngeal, subcarinal, left gastric, paracardiac, upper paraesophageal, and hepatogastric—were free of metastasis. The patient received postoperative supportive care, including antibiotics, acid suppression, mucolytics, antispasmodics, intravenous nutrition, and albumin supplementation. On postoperative day 7, iodine water angiography revealed no significant abnormalities at the anastomosis, permitting the reintroduction of a liquid diet. The patient was discharged in stable condition on postoperative day 10. No evidence of progression or recurrence has been observed during the follow-up. This report aims to inform clinical practice with insights into the management of rare coexisting pathologies

## Background

**Figure 1:**
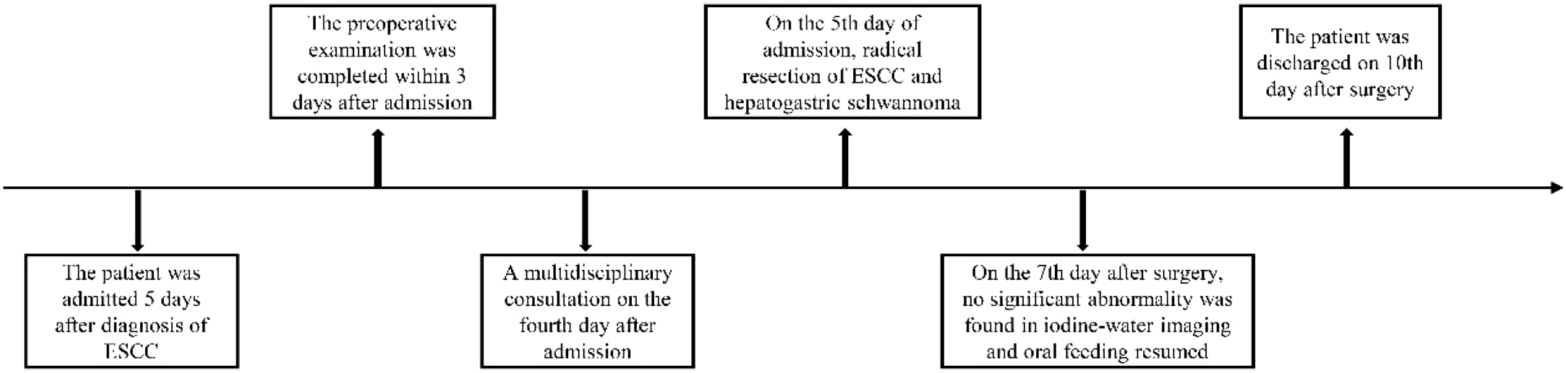
Timeline of patient treatment.

Esophageal cancer is a common malignancy of the digestive tract and remains a leading cause of cancer-related deaths worldwide (1–3). Its incidence and mortality rates vary across countries, with China reporting one of the highest burdens (4). Progressive dysphagia is the hallmark symptom, initially manifesting as difficulty swallowing dry or hard foods, later advancing to semi-liquid foods. Early detection through gastroscopy is crucial, as it allows timely intervention and improved outcomes via comprehensive treatment, primarily surgery, supplemented by immunotherapy, chemotherapy, and radiotherapy. This report presents a case of esophageal squamous cell carcinoma (ESCC) identified during a routine health check-up. Enhanced upper abdominal computed tomography (CT) scan revealed a 3.3 × 2.6 cm mass in the hepatogastric space, with mildly enlarged surrounding lymph nodes, the largest measuring 0.8 cm in short diameter, indicating possible metastasis. Endoscopic ultrasonography indicated early-stage esophageal cancer. Multidisciplinary team (MDT) evaluation concluded that hepatogastric lymph node metastasis was unlikely and that the mass was more likely benign. Therefore, the patient and family opted for robot-assisted radical resection of ESCC. On postoperative day 7, iodine water angiography revealed no abnormalities at the anastomosis, and an oral liquid diet was resumed. The patient was discharged in good condition on postoperative day 10 (**Figure 1**). This case aims to offer clinical insights and treatment strategies for managing similar conditions.

## Case description

**Figure 2:**
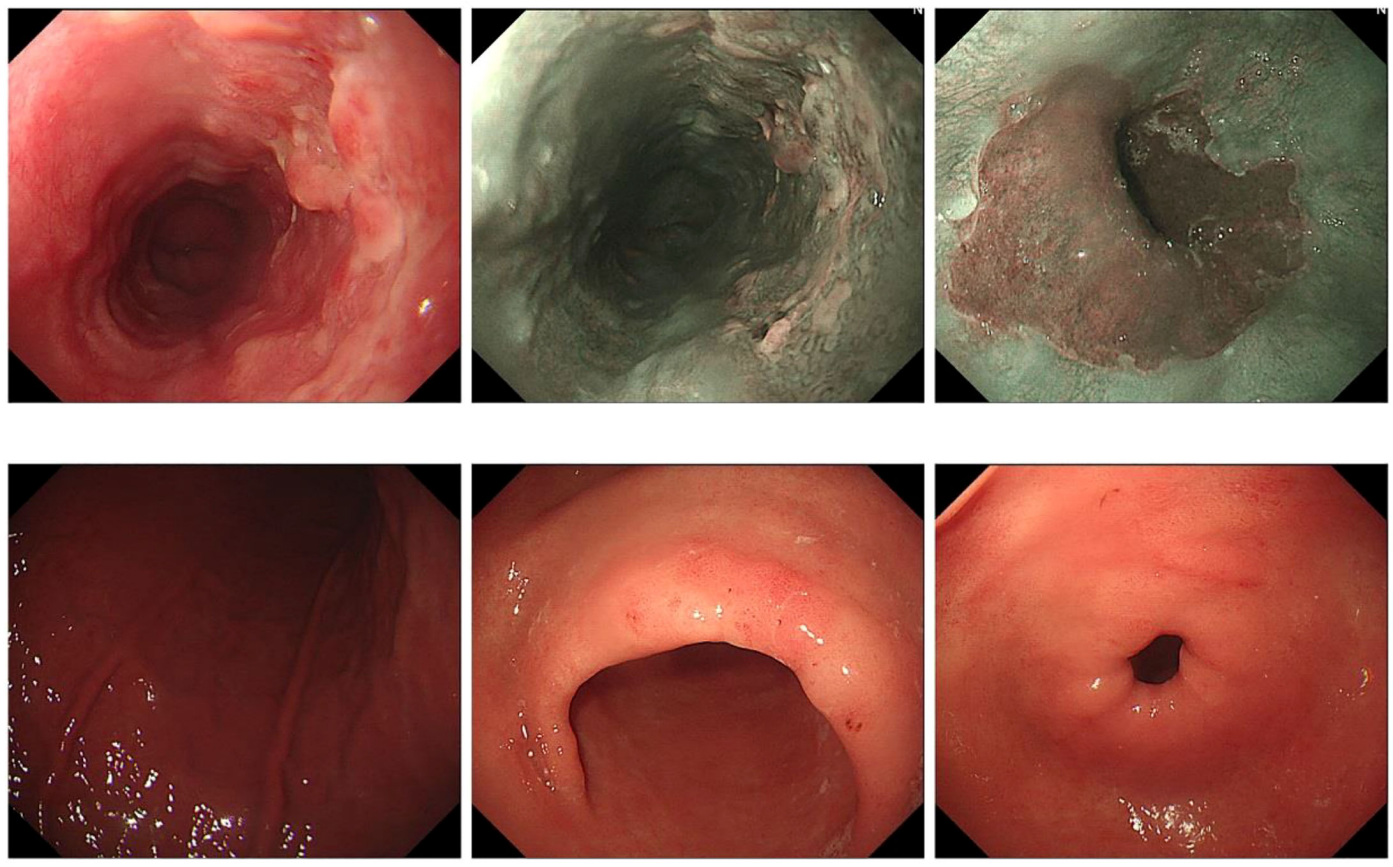
Esophageal lesions observed via gastroscopy.

A 69-year-old male patient from Shiyan City underwent gastroscopy at our hospital 5 days prior, revealing a raised erosive lesion in the esophagus, approximately 32–38 cm from the incisor. Pathology confirmed high-grade intraepithelial neoplasia of esophageal squamous epithelium and squamous cell carcinoma formation (**Figure 2**). At diagnosis, the patient reported no dysphagia, abdominal pain, bloating, nausea, vomiting, or other gastrointestinal discomfort, nor respiratory or systemic symptoms such as cough, sputum, palpitations, dyspnea, fever, or weight loss. His medical history included an insufficient cerebral blood supply with intermittent dizziness and malaise. The patient had a history of prostatic hyperplasia for over 2 weeks, with intermittent difficulty in urination. His family had a history of esophageal cancer, and shared similar dietary habits. He reported no chronic conditions such as hypertension, diabetes, or cardiovascular disease. There was no history of previous surgeries, trauma, or known food or drug allergies.

**Figure 3:**
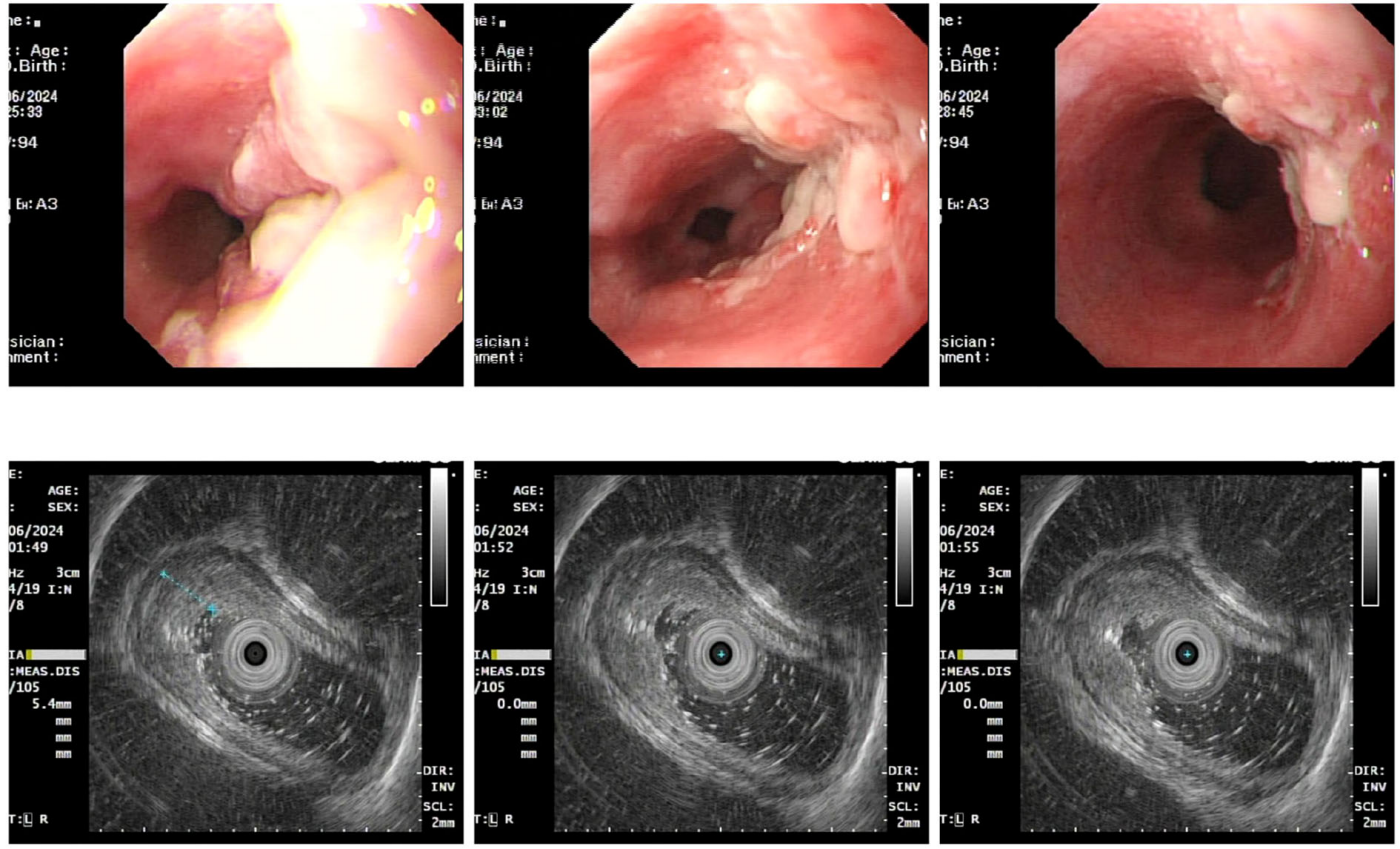
Esophageal lesions visualized under endoscopic ultrasonography.

**Figure 4:**
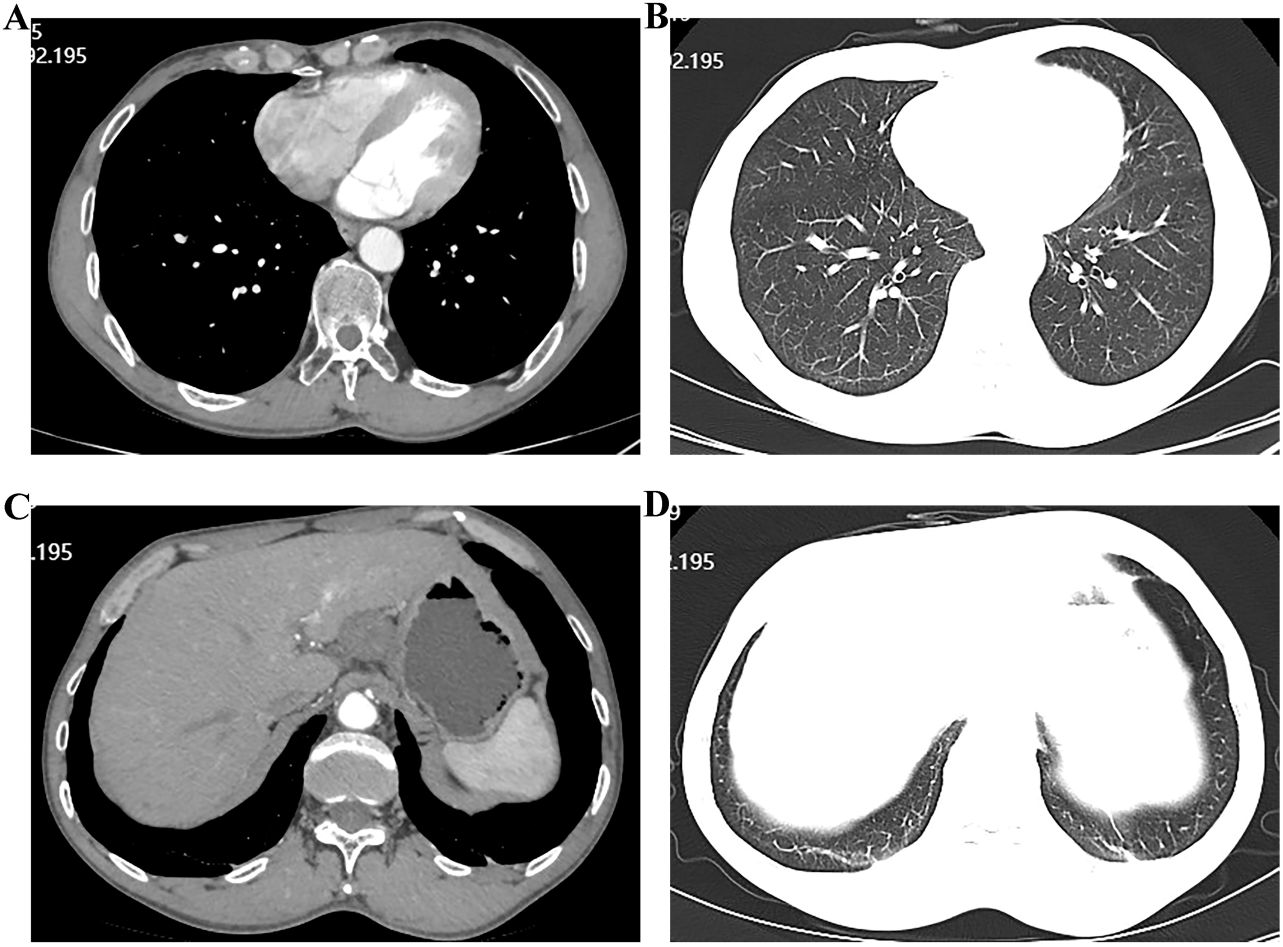
Computed tomographic (CT) images showing esophageal and hepatic gastrointestinal space-occupying lesions. **(A, B)** esophageal lesion; **(C, D)** Hepatic gastrointestinal space-occupying lesion.

Upon admission, diagnostic investigations were completed. Endoscopic ultrasonography revealed a lesion with moderate-to-low echogenicity involving the muscularis propria (**Figure 3**). Contrast-enhanced chest CT showed mild thickening of the lower esophageal wall, suggesting possible lower esophageal carcinoma (**Figures 4A, B**). Abdominal contrast-enhanced CT revealed a 3.3 × 2.6 cm mass in the hepatogastric region with mildly enlarged lymph nodes, the largest measuring approximately 0.8 cm in the short axis, raising suspicion of metastasis (**Figures 4C, D**). Coronary CT angiography (CTA) revealed a myocardial bridge in the mid-left anterior descending artery (LAD). Cranial CT suggested cerebral atrophy, and head and neck CTA indicated carotid arteriosclerosis. Cardiac ultrasound revealed mild mitral and tricuspid regurgitation. Venous doppler of the lower limbs showed no evidence of deep vein thrombosis. A multidisciplinary team evaluated the hepatogastric mass and advised preoperative nutritional optimization. After thorough preoperative preparation, the patient underwent robot-assisted thoracoscopic partial esophagostomy, intrathoracic esophagogastrostomy, thoracoscopic adhesiolysis, mediastinal lymphadenectomy, and excision of the hepatogastric mass on July 23, 2024, under general anesthesia.

### Surgical procedure

General anesthesia was successfully induced with single-lumen endotracheal intubation. The patient was positioned in a slight left lateral decubitus position. After standard disinfection, four incisions (0.8-1.2 cm) were made: at the third intercostal space (right midaxillary line), sixth intercostal space (posterior axillary line), fifth intercostal space (anterior axillary line), and ninth intercostal space (between the posterior axillary and scapular lines). No pleural effusion was noted upon exploration. After releasing thoracic adhesions, the azygos vein was mobilized and transected using a linear stapler. The thoracic esophagus was carefully dissected from the thoracic inlet to the esophageal hiatus using a robotic electrosurgical hook, bipolar forceps, and ultrasonic scalpel. Systematic lymphadenectomy included nodes beneath the tracheal bifurcation and along both recurrent laryngeal nerves. After confirming the absence of pulmonary air leaks, the robotic system was withdrawn, and a chest tube was placed through the sixth intercostal space at the posterior axillary line. The patient was then repositioned supine. Following standard disinfection and draping, 0.5-1.2 cm incisions were made along the left and right anterior axillary lines below the umbilicus, approximately 2 cm left of the umbilicus, above the costal arch at the left axillary line, and below the right rectus abdominis adjacent to the costal margin. Under artificial pneumoperitoneum, a robotic ultrasonic scalpel was employed to mobilize the greater curvature of the stomach, manage the short gastric vessels, and preserve the right gastroepiploic artery. The lesser omental sac was opened to mobilize the lesser curvature of the stomach. A well-circumscribed, rounded mass approximately 3 cm in diameter was identified above the left gastric artery. It was separated from surrounding tissues and excised using Maryland forceps for routine histopathology. After double clamping with Hem-o-lock clips, the left gastric artery was transected. Following esophageal hiatus mobilization, the robotic system was removed. A 6 cm cervical incision was made along the anterior border of the left sternocleidomastoid muscle, extending to the esophageal space. The esophagus was transected, and a gastric tube was tied to the distal esophageal stump for traction. The subxiphoid incision was expanded to 5 cm, allowing extraction of the stomach, esophagus, and associated perigastric lymph nodes and masses. A tubular stomach was fashioned, and its blind end was ligated and sutured to the esophageal stump. An end-to-end anastomosis was performed using a three-layer technique for the posterior wall and a two-layer technique for the anterior wall. The gastric tube was positioned approximately 20 cm from the incisors, and a rubber drain was placed in the cervical incision. All abdominal and cervical incisions were subsequently closed.

**Figure 5:**
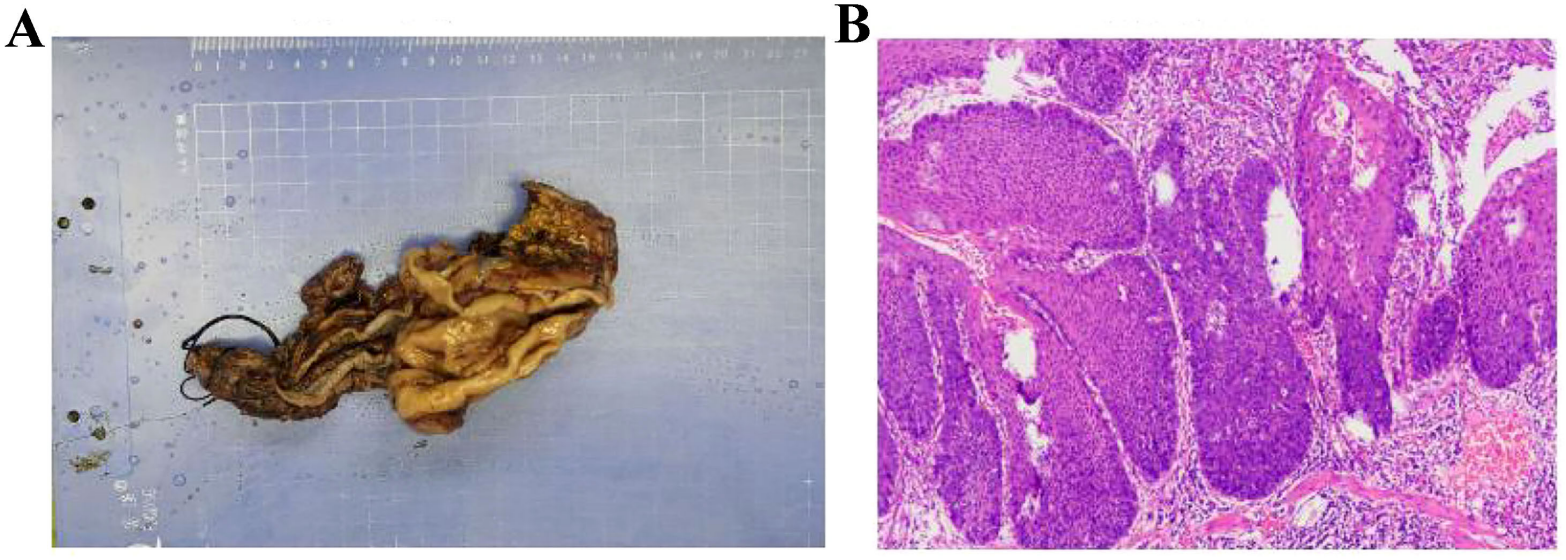
Postoperative pathology confirming esophageal squamous cell carcinoma. **(A)** Specimen **(B)** Pathological sections.

Pathological examination confirmed pT1aN0M0 poorly to moderately differentiated esophageal squamous cell carcinoma (**Figure 5**). The tumor measured 3.0 cm × 2.7 cm × 0.2 cm, confined to the mucosa without vascular or perineural invasion. Margins at the gastric incisal edge and the upper anastomotic stump were free of malignancy. No metastases were found in the perigastric (0/5), paracardiac (0/1), left recurrent laryngeal (0/2), upper paraesophageal (0/2), left gastric (0/2), or hepatogastric lymph nodes (0/3). The right recurrent laryngeal and subcarinal lymph nodes contained only fibro-adipose tissue microscopically. The mass, measuring 3.5 cm in diameter and located between the liver and stomach, was pathologically identified as a schwannoma. Postoperative management included cefradine for infection control, along with acid suppression, mucolytics, antispasmodics, and albumin supplementation.

**Figure 6:**
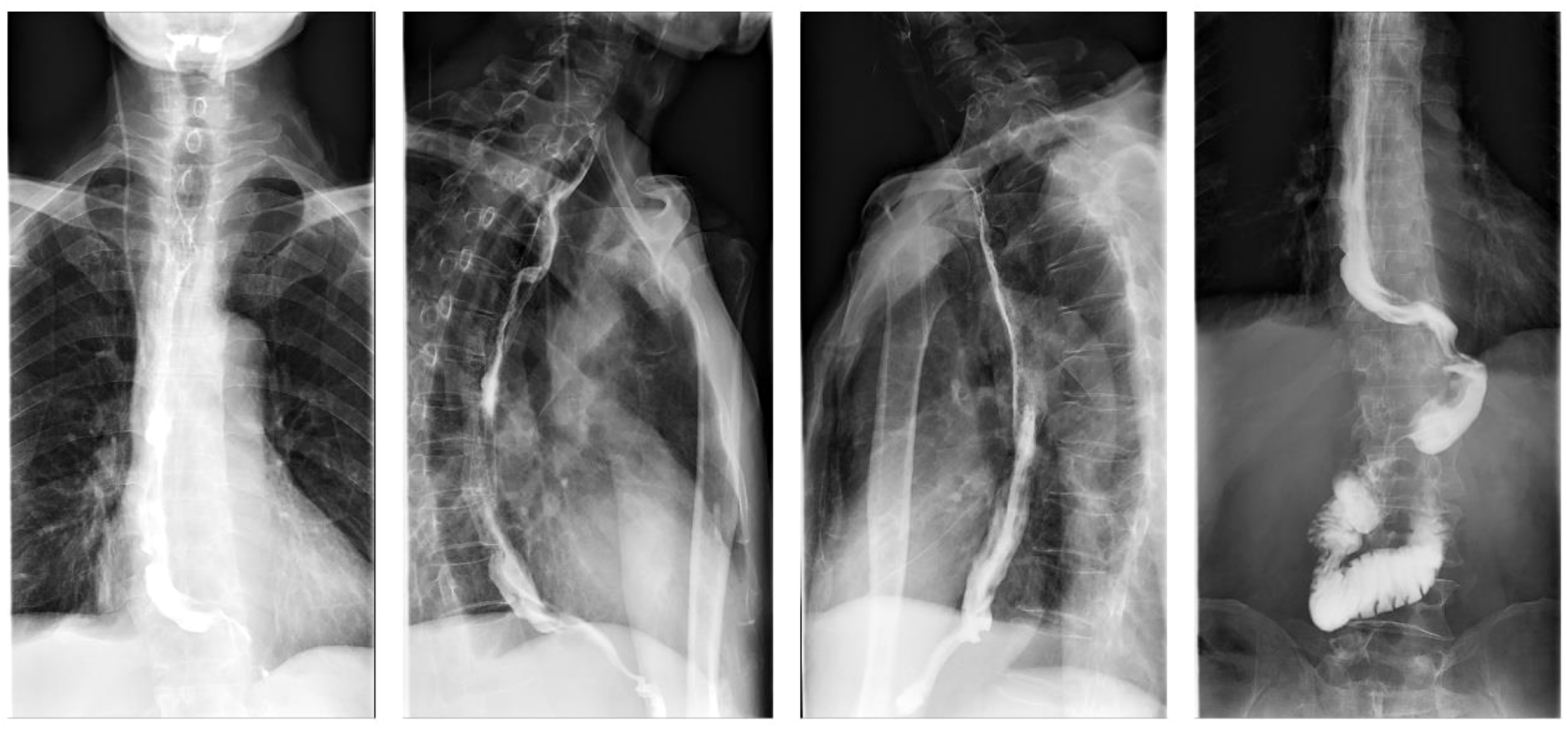
Esophageal iodine contrast study showing postoperative anatomical changes.

**Figure 7:**
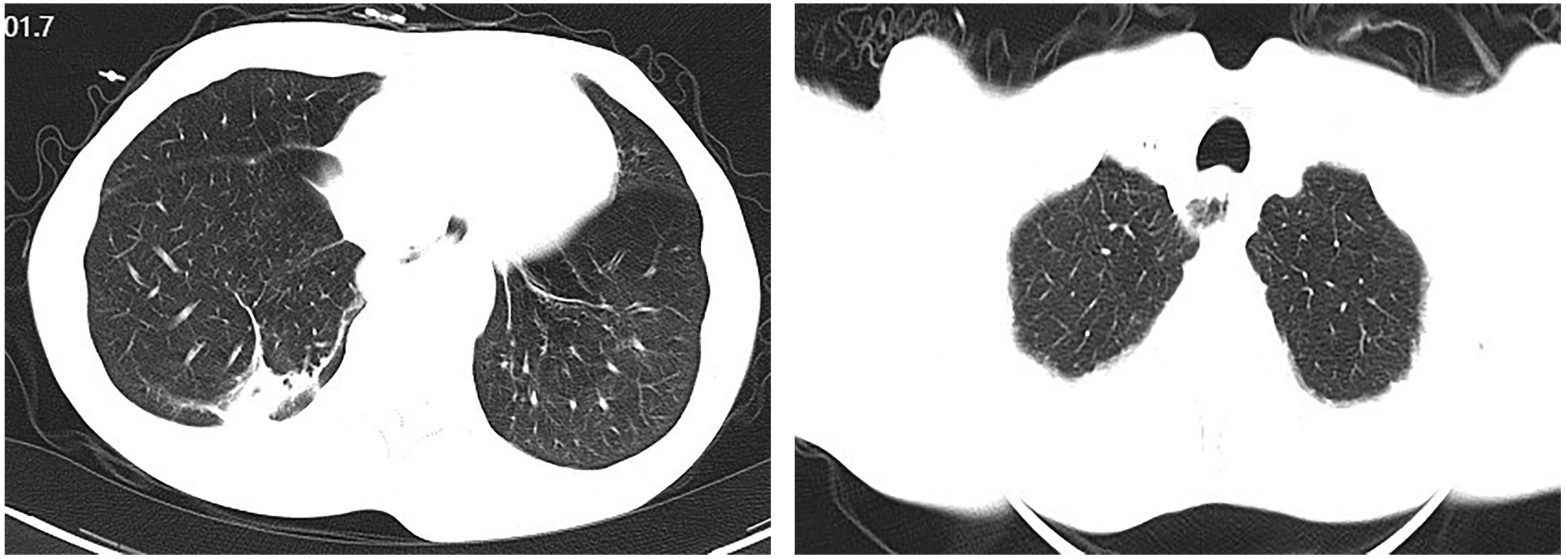
Chest computed tomographic (CT) demonstrating postoperative changes in the esophagus.

On postoperative day 7, gastrointestinal iodine-water angiography revealed no evidence of significant stenosis or leakage at the esophagogastric anastomosis (**Figure 6**). Upon resuming a liquid diet, the patient reported no discomfort, including fever, chest pain, or chest tightness. The chest CT scan showed no significant abnormalities (**Figure 7**). The patient was stable and discharged on postoperative day 10. At approximately 10 months post-discharge, telephone follow-up indicated no signs of disease progression or recurrence.

## Discussion

Esophageal cancer has a high incidence in China, which also ranks among the countries with the highest mortality rates from this disease (4, 5). There are also substantial regional variations in incidence across the country (5). The histological types of esophageal cancer are classified as esophageal squamous cell carcinoma and esophageal adenocarcinoma, with squamous cell carcinoma accounting for over 90% of cases in China. The hallmark clinical symptom is progressive dysphagia, though early-stage disease is frequently detected incidentally through gastroscopy. Early-stage esophageal cancer can be effectively treated with endoscopic procedures or surgical resection. However, many patients seek medical attention only after experiencing significant dysphagia, by which time the disease has typically advanced. These patients frequently require multimodal therapy, including immunotherapy, chemotherapy, radiotherapy, and supportive care, to improve outcomes (6–10).

In this case, the patient had a familial history of esophageal malignancy—his elder brother was diagnosed over four years prior. Therefore, the patient requested a gastroscopy during a routine health check-up, which identified an elevated erosive lesion located approximately 32–38 cm from the incisors. Histopathology revealed high-grade intraepithelial neoplasia of the squamous epithelium, indicative of squamous cell carcinoma. As the patient exhibited no meal-related obstructive symptoms, early-stage esophageal cancer was suspected. However, contrast-enhanced abdominal CT revealed a 3.3 × 2.6 cm mass in the hepatogastric space with mildly enlarged adjacent lymph nodes, the largest measuring approximately 0.8 cm in short-axis diameter, raising concern for potential metastasis. Given the patient’s clinical history and imaging features, the hepatogastric mass was considered more likely benign. After multidisciplinary consultation, the patient and the family opted for surgical treatment. Robotics surgery, now routinely used in minimally invasive procedures for colorectal and esophageal cancers (11, 12), was selected. As the patient had not received neoadjuvant therapy, robot-assisted thoracoscopic partial esophagectomy, intrathoracic esophagogastric anastomosis, thoracoscopic adhesiolysis, and mediastinal lymphadenectomy were performed under general anesthesia on July 3, 2024. The procedure was completed successfully without complications. Postoperative pathology confirmed early esophageal cancer, with no evidence of metastasis in mediastinal or abdominal lymph nodes. The hepatogastric mass was diagnosed as a schwannoma.

Schwannomas, also known as neurolemmomas, are benign tumors that arise from the sheath surrounding peripheral nerves. They most commonly affect the vestibular or cochlear nerves. However, their occurrence in the hepatogastric space is rare and typically requires histopathological examination for confirmation. In this case, the hepatogastric schwannoma could not be biopsied preoperatively, making a definitive preoperative diagnosis unfeasible. Following robot-assisted thoracoscopic partial esophagectomy, intrathoracic esophagogastric anastomosis, thoracoscopic adhesiolysis, and mediastinal lymphadenectomy, pathological diagnosis confirmed the preoperative suspicion of schwannoma.

Anastomotic leakage is the most frequent complication following radical esophagectomy. In our center, an end-to-end anastomosis technique was employed. On postoperative day 7, gastrointestinal iodine-water angiography revealed no significant stenosis or leakage at the anastomosis site. The patient resumed a liquid diet without experiencing fever, chest pain, or shortness of breath. On postoperative day 10, the patient was stable and discharged in good condition. This case highlights a rare presentation of hepatogastric schwannoma and demonstrates the successful use of robotic-assisted esophagectomy. Reporting this case aims to inform clinical practice and inspire optimism for the management of other patients with esophageal cancer.

## Data Availability

The original contributions presented in the study are included in the article/Supplementary Material. Further inquiries can be directed to the corresponding authors.
